# HELIOS-expressing human CD8 T cells exhibit limited effector functions

**DOI:** 10.3389/fimmu.2023.1308539

**Published:** 2023-12-22

**Authors:** Damien Neyens, Thibault Hirsch, Achraqat Abdel Aziz Issa Abdel Hadi, Nicolas Dauguet, Christophe Vanhaver, Alexandre Bayard, Claude Wildmann, Mathieu Luyckx, Jean-Luc Squifflet, Quentin D’Hondt, Céline Duhamel, Antoine Huaux, Virginie Montiel, Mélanie Dechamps, Pierre van der Bruggen

**Affiliations:** ^1^ De Duve Institute, Université Catholique de Louvain, Brussels, Belgium; ^2^ Département de gynécologie, Cliniques Universitaires Saint-Luc, Brussels, Belgium; ^3^ Unité de soins intensifs, Cliniques Universitaires Saint-Luc, Brussels, Belgium; ^4^ Walloon Excellence in Life Sciences and Biotechnology (WELBIO), Wavre, Belgium

**Keywords:** HELIOS, CD8 T lymphocytes, Tc17, HLA-E, human

## Abstract

**Introduction:**

The transcription factor HELIOS is primarily known for its expression in CD4 regulatory T cells, both in humans and mice. In mice, HELIOS is found in exhausted CD8 T cells. However, information on human HELIOS^+^ CD8 T cells is limited and conflicting.

**Methods:**

In this study, we characterized by flow cytometry and transcriptomic analyses human HELIOS^+^ CD8 T cells.

**Results:**

These T cells primarily consist of memory cells and constitute approximately 21% of blood CD8 T cells. In comparison with memory HELIOS^-^ T-BET^high^ CD8 T cells that displayed robust effector functions, the memory HELIOS^+^ T-BET^high^ CD8 T cells produce lower amounts of IFN-γ and TNF-α and have a lower cytotoxic potential. We wondered if these cells participate in the immune response against viral antigens, but did not find HELIOS^+^ cells among CD8 T cells recognizing CMV peptides presented by HLA-A2 and HLA-B7. However, we found HELIOS^+^ CD8 T cells that recognize a CMV peptide presented by MHC class Ib molecule HLA-E. Additionally, a portion of HELIOS^+^ CD8 T cells is characterized by the expression of CD161, often used as a surface marker for identifying T_C17_ cells. These CD8 T cells express T_H17_/T_C17_-related genes encoding RORgt, RORa, PLZF, and CCL20.

**Discussion:**

Our findings emphasize that HELIOS is expressed across various CD8 T cell populations, highlighting its significance beyond its role as a transcription factor for Treg or exhausted murine CD8 T cells. The significance of the connection between HELIOS and HLA-E restriction is yet to be understood.

## Highlights

- HELIOS^+^ CD8 T cells produce low amounts of IFN-γ.
- HELIOS is expressed by a subset of viral-specific CD8 T cells restricted by HLA-E and not by HLA-class Ia-restricted CD8 T cells.

## Introduction

T cell identity and functions are governed by transcription factors. One such transcription factor, HELIOS, is encoded by the IKAROS Zinc finger protein 2 gene (*IKZF2*). Previous studies have primarily focused on the roles of HELIOS in regulatory CD4 T cells (T_regs_), both in humans and mice. HELIOS was also reported to be expressed in exhausted CD8 T cells in mice (1–10).

Approximately 70% of murine and human CD4 T_regs_ express HELIOS, which is usually described as a marker distinguishing thymic-derived from peripherally induced CD4 T_regs_ (5, 8, 9). While HELIOS is necessary to maintain the T_reg_ phenotype, its role is less prominent than FOXP3. In *Ikzf2*-KO mice, autoimmune symptoms develop after 6 to 8 months, in contrast to FOXP3-deficient (scurfy) mice that succumb to the disease within weeks (2, 4). HELIOS^+^ CD4 T_regs_ suppress T cell functions more effectively than HELIOS^-^ CD4 T_regs_ (7, 10). Moreover, engineered Tregs that overexpress both FOXP3 and HELIOS demonstrate better immunosuppressive properties (11).

In mice, HELIOS expression is upregulated in exhausted CD8 T cells during chronic LCMV infection (1, 3, 6). These exhausted CD8 T cells exhibit reduced functionality compared to effector CD8 T cells, such as lytic activity and production of IFN-γ and TNF-α (12, 13). CD8 T cells from mice with dysregulated NFAT1-AP1 signalling share common characteristics with exhausted CD8 T cells, such as increased expression of PD-1, TIM-3, LAG-3 and TOX, and upregulate expression of HELIOS (14).

In human blood, 15% of CD8 T lymphocytes have been found to express HELIOS (15). While some authors have suggested that HELIOS is a marker of T cell activation due to increased levels observed in proliferating T cells *in vitro*, others have failed to reproduce these observations (16). For instance, HELIOS was not expressed by CD8 T cells specifically targeting the HLA-A2-restricted epitope of the tick-borne encephalitis virus (TBEV) during the immune response (15). Furthermore, activated Ki-67^+^ CD38^+^ CD8 T cells were found to express less HELIOS than resting Ki-67^-^ CD38^-^ CD8 T cells (15), contradicting the previous observation (16).

A heterozygous loss-of-function variant in IKZF2 has been described in a single family, leading to immunodeficiency with increased immune activation and a profound reduction in mucosal-associated invariant T (MAIT) cells (17). Patients carrying the IKZF2 variant presented with a combined immunodeficiency phenotype characterized by recurrent upper respiratory infections, thrush, mucosal ulcers, and chronic lymphadenopathy. Reduced Helios expression was associated with chronic T cell activation and increased production of proinflammatory cytokines in both effector and regulatory T cells (17).

During a preliminary experiment, we noticed the presence of HELIOS^+^ CD8 T cells in human blood of donors that did not produce high amounts of IFN-γ. This finding was intriguing considering the expression of HELIOS in murine exhausted CD8 T cells, murine CD4 and CD8 Tregs, as well as human CD4 T_regs_ (1, 3, 4, 6, 9). Besides, information regarding HELIOS^+^ in human CD8 T cells remains limited and contradictory. Therefore, we have undertaken a study to investigate this HELIOS^+^ CD8 T cell population in the blood of donors, aiming to define its characteristics and gain insights into its potential role.

## Materials and methods

### T cell culture

T cells were cultured in Iscove’s Modified Dulbecco’s Medium (IMDM) (Thermo Fischer Scientific, Waltham, Massachusets, 21980065) supplemented with 10% human serum (HS) (Ludwig Institute for Cancer Research (LICR), Brussels branch), GlutaMAX (Thermo Fischer Scientific, 35050061) and penicillin (100U.ml^-1^) streptomycin (100μg.ml^-1^) (Sigma Aldrich, St Louis, Missouri, P4333) referred to as T cell medium.

### Isolation of cells from blood and tumors

Patients gave written informed consent, and their records were anonymized prior to the analysis. The cohorts of patients are described in **Supplementary Tables 1–3**. Peripheral blood mononuclear cells (PBMCs) were isolated from blood samples obtained from healthy, hemochromatosis, COVID-19 and ovarian cancer patients using a density gradient. Blood was added on top of lymphoprep (Alere Technologies, Oslo, Norway, 1114547) and centrifuged at 870g for 20min at room temperature (RT) with minimum acceleration and brake. The cellular ring was recovered and washed thrice with phosphate buffer saline (PBS)/EDTA 1mM. First centrifugation was done at 400g for 10min at RT and the two following at 300g for 7min at RT. PBMCs were frozen in 50% IMDM 40% human serum (HS) and 10% Dimethylsulfoxide (DMSO) (Santa Cruz Biotechnology, Dallas, Texas, sc-358801). Cells from ovarian tumors were isolated by mechanical dissociation using a MACS dissociator (Miltenyi, Bergisch Gladbach, Germany) followed by a 45min enzymatic digestion at 37°C using liberase low dispase (Sigma Aldrich, 5466202001) and low thermolysin (Sigma Aldrich, 5401020001) at recommended dilutions. A second mechanical dissociation was performed and the subsequent cell suspension was 40μm filtered. Cells were then washed thrice with PBS/EDTA 1mM, spun down at 300g for 7min at RT and frozen in 50% IMDM 40% HS and 10% DMSO.

### Cytokine production assay

High binding 96-well flat bottom plates (Greiner bio-one, 655061) were coated with 100μl of 1μg/ml anti-CD3 antibody (Biolegend, San Diego, California, clone OKT3, 317326) diluted in PBS overnight at 4°C. Human PBMCs were thawed for 2h at 37°C in T cell medium with 5U/ml DNAse. Anti-CD3 coated plates were brought at 37°C for half an hour before stimulation. A total of 2x10^5^ cells were added per well in T cell medium. Cells were spun down 1min at 400g, and incubated at 37°C 8% CO2. After 1 hour, 5μg/ml brefeldin A (Sigma Aldrich, B7651) were added and cells were incubated for an additional 4h at 37°C 8% CO2. After a total of 5h (1h+4h) of stimulation, cells were brought to 4°C and stained as described in the flow cytometry section. Alternatively, the cells were stimulated with PMA (1ng/ml; LC laboratories #P-1680) and ionomycin (1 µg/ml, Sigma-Aldrich@I0634)) using the same protocol as described for stimulation with anti-CD3.

### TNF Secretion

High binding 96-well flat bottom plates were coated with 100ml of 1mg/ml anti-CD3 antibody diluted in PBS overnight at 4°C. Human PBMCs were thawed for 2h at 37°C in T cell medium with 5U/ml DNAse. Anti-CD3 coated plates were brought at 37°C for half an hour before stimulation. A total of 2x105 cells were added per well in T cell medium containing 10mg/ml TAPI-0 (Sigma, SML1292) and 2mg/ml anti-TNF-a Alexa Fluor 700 antibody (Biolegend, clone Mab11, 502928) and spun down 400g for 1min before incubation at 37°C 8% CO2 for 5h. Next, cells were brought to 4°C and stained as described in the flow cytometry section. 

### Flow cytometry

For multimer staining, cells were harvested, washed once with staining buffer (PBS, 1mM EDTA and 1% HS) and then labeled with 10nM phycoerythrin (PE)-multimer for 10min at RT (**Table 1**). After, cells were diluted with one volume of staining buffer and incubated 15min at RT. Cells were washed twice with staining buffer at 4°C and spun down at 400g. Next, extracellular and intracellular staining with antibodies and fixable viability dye was performed as described below.

**Table 1:** List of multimers used.

Origin	Protein	Peptide	HLA	Fluorochrome
CMV	pp65	NLVPMVATV	HLA-A*02:01	PE
CMV	pp65	RPHERNGFTVL	HLA-B7*07:02	PE
CMV	pp65	TPRVTGGGAM	HLA-B7*07:02	PE
CMV	UL-40	VMAPRTLIL	HLA-E*01:01	PE
EBV	BMFL-1	GLCTLVAML	HLA-A*02:01	PE
EBV	BZLF-1	SQAPLPCVL	HLA-E*01:03	PE
Influenza	M1	GILGFVFTL	HLA-A*02:01	PE
Mycobacterium tuberculosis	Rv1508	VMATRRNVL	HLA-E*01:01	PE

For extracellular and intracellular staining, cells were harvested, washed once with staining buffer and then labeled extracellularly for 20min at 4°C with antibodies and fixable viability dye eFluor780 (Thermo Fischer Scientific, 65-0865-18) (**Table 2**). Cells were washed twice with staining buffer and once with PBS. Then, cells were fixed and permeabilized overnight at 4°C using eBioscience Foxp3/Transcription Factor kit (Thermo Fisher Scientific, 00-5523-00). Cells were washed thrice with permeabilization buffer at 4°C, resuspended in blocking solution (permeabilization buffer 8% HS) and incubated for 15min at 4°C. Next, antibodies, diluted in the permeabilization buffer from the kit, were added for 2h at 4°C (**Table 3**). Cells were washed thrice with permeabilization buffer. Finally, cells were resuspended in PBS 1% PFA and analyzed by flow cytometry using BD LSRII Fortessa (Becton Dickinson (BD) Biosciences, Franklin Lakes, New Jersey). Data were analyzed using Flowjo version 9 (BD Biosciences).

**Table 2:** List of antibodies used for extracellular staining.

Target	Fluorochrome	Clone	Concentration	Reference
CD19	APC-Cy7	SJ25C1	1μg/ml	*Biolegend, 363010*
CD20	APC-Cy7	2H7	1μg/ml	*Biolegend, 302314*
CD33	APC-Cy7	WM53	1μg/ml	*Biolegend, 303442*
CD326	APC-Cy7	9C4	1μg/ml	*Biolegend, 324246*
CD2	BV480	RPA-2.10	0.4μg/ml	*BD Biosciences, 746538*
CD8β	PerCP-Cy5.5	2ST8.5H7	2μg/ml	*BD Biosciences, custom*
CCR7	PE	G043H7	2μg/ml	*Biolegend, 353204*
CCR7	BV650	G043H7	2.5μg/ml	*Biolegend, 353234*
CD45RA	FITC	HI100	2.5μg/ml	*Biolegend, 304148*
PD-1	BV786	EH12.2H7	2.5μg/ml	*Biolegend, 329930*
CD38	BV480	HIT2	10μg/ml	*BD Biosciences, 566137*

**Table 3:** List of antibodies used for intracellular staining.

Target	Fluorochrome	Clone	Concentration	Reference
Ki-67	BV421	B56	0.5μg/ml	*BD Biosciences, 562899*
HELIOS	PE-Cy7	22F6	0.12μg/ml	*Biolegend, 137236*
HELIOS	PE	22F6	0.12μg/ml	*Biolegend, 137216*
HELIOS	APC	22F6	0.2μg/ml	*Biolegend, 137222*
T-BET	BV421	4B10	1μg/ml	*Biolegend, 644816*
T-BET	APC	4B10	1μg/ml	*Biolegend, 644814*
IFN-γ.	Alexa-Fluor 700	B27	3μg/ml	*Biolegend, 506516*
TNF-α.	Alexa-Fluor 700	Mab11	1μg/ml	*Biolegend, 502928*
IL-2	PE	MQ1-17H12	1μg/ml	*Biolegend, 500307*
Histone H1	Alexa Fluor 488	AE-4	4μg/ml	*Santa Cruz Biotechnology, sc-8030 AF488*
Histone H1	Alexa Fluor 647	AE-4	4μg/ml	*Santa Cruz Biotechnology, sc-8030 AF647*

Particularities for intracellular staining followed by RNA extraction: when cells were stained intracellularly and FACS sorted for RNA extraction, RNAse inhibitor RNAsin Plus *(Promega, Madison, Wisconsin, N2615)* was added during the fixation/permeabilization overnight at 2U/μl. During washes, RNAsin Plus was added at 0,04U/μl. During the staining, RNAsin Plus was at 2U/μl and at the end, cells were not resuspended in PFA 1% in this case but in the permeabilization buffer containing 0,4U/μl RNAsin Plus. During the FACS sorting, cells were recovered in a permeabilization buffer containing 0,04U/μl RNAsin Plus.

### RNA extraction from fixed and permeabilized cells

Recover all total nucleic acid isolation kit (Thermo Fisher Scientific, AM1975) was used. The reverse crosslinking with protease was done for 1h at 60°C. Then, RNA was extracted using the manufacturer protocol. After elution in water, RNA was precipitated by adding 0,1 volume sodium acetate 3M (Thermo Fischer Scientific, AM9740), 3 volumes ice-cold ethanol 100% and 1μg glycogen (Thermo Fisher Scientific, R0551). Samples were incubated at -20°C overnight and spun down at 14000g 30min at 4°C. Samples were then washed twice with ice-cold ethanol 75% and spun down at 14000g 10min at 4°C. After supernatant removal, samples were dried at RT and then resuspended in RNAse-free water.

### RNA-sequencing and transcriptomic analysis

Libraries were homemade using the SMART-Seq2 protocol of Picelli et al. (18). We, however, used biotinylated oligos and smaller reaction volumes (3/5) for the reverse transcription and PCR preamplification steps. Briefly, 1.5μl RNA was retrotranscribed for 90 minutes at 42°C followed by 10 cycles of 2 minutes at 50°C and 2 minutes at 42°C in the presence of 10U/μl SuperScript II reverse transcriptase (Thermo Fisher, #18064071), 1μM oligo-dT and 1μM oligo template switch (TSO) (Eurogentec). The resulting cDNA was amplified over 14 cycles of 20 seconds at 98°C, 15 seconds at 67°C and 6 minutes at 72°C, in the presence of 0.02U/μl KAPA HiFi HotStart DNA polymerase (Roche, #07958897001) and 100nM oligo IS PCR (Eurogentec). Amplified cDNA was purified with 0.9 volume of AMPure XP paramagnetic beads (Analis, #A63881) for 1 volume of sample, then assayed with the QuantiFluor dsDNA System kit (Promega, #E2670). DNA was tagged for 5 minutes at 55°C in the presence of Tn5 transposase and the primers supplied with the tagging kit (Illumina, #FC-131-1096). The tagged DNA was then amplified over 11 cycles of 10 seconds at 95°C, 30 seconds at 55°C, and 30 seconds at 72°C in the presence of the indexed primers supplied with the kit (Illumina, #FC-131-2004). The amplified and indexed product was purified with 0.8 volume of AMPure XP paramagnetic beads per 1 volume of sample, then assayed with the QuantiFluor dsDNA System kit. Concentrations were normalized and an equal quantity of each sample was pooled to obtain an equimolar library of up to 16 samples pooled together. The resulting library size was verified by capillary electrophoresis on the TapeStation (Agilent, #5067-5588, #5067-5589). The libraries were sequenced by Genewiz on the Illumina HiSeq platform. Approximately 380.10^6^ pairs of 150bp reads per sequencing line were obtained, which means around 20-25 million pairs of 150bp reads per sample. In brief, after initial quality control of FASTQ files, reads were trimmed using Trimmomatic and checked again for quality (19). Next, trimmed reads were aligned to GRCh38 human genome with HISAT2 (20). Read counts were determined with featuresCount (21). Normalization of read counts and analysis of differentially expressed genes were performed using DESeq2 (22). Data visualization was done with Qlucore Omics Explorer (Qlucore AB, Lund, Sweden).

### Statistical analyses

Statistical analyses were made with GraphPad Prism v5 (GraphPad, San Diego, California) except RNA-sequencing for which paired samples analysis was done with DESeq2 and ANOVA analysis with Qlucore Omics Explorer. Statistical tests are indicated in the figure legend.

## Results

### Most HELIOS^+^ CD8 T cells are antigen-experienced and are activated during cancer and viral infection

**Figure 1:**
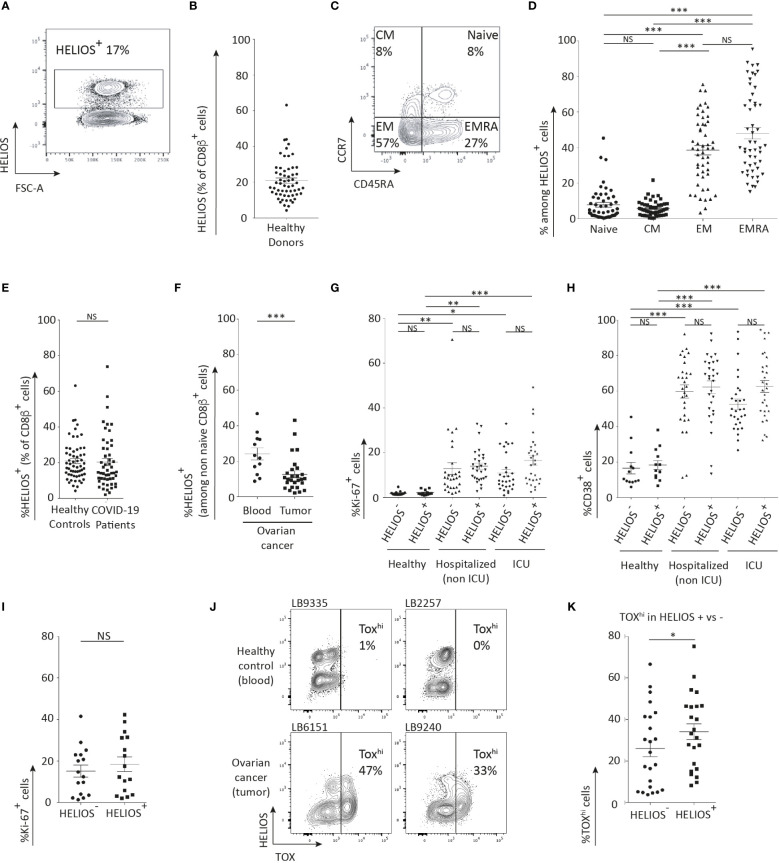
The majority of HELIOS^+^ CD8 T cells are antigen-experienced. Frozen PBMC were thawed and rested for 2h at 37°C with DNase. Additionally, CD38 was labeled for healthy donors and COVID-19 patients. Ovarian cancer patient samples were also labeled with CD19, CD20, CD33, and CD326. Following overnight fixation and permeabilization, intracellular staining for HELIOS was performed at 4°C. Ki-67 staining was also performed for healthy donors and COVID-19 patients. The samples were analyzed by flow cytometry. **(A)** Representative donor’s data showing the prevalence of HELIOS+ cells within living CD2^+^ CD8β^+^ cells **(B)** data for 58 donors. These data were obtained from 13 females and 45 males. The raw data are also available in **Supplementary Table 1**. The mean frequencies of HELIOS^+^ cells were 26% and 20% respectively. These two mean frequencies are not different according to an unpaired t-test. **(C)** Distribution of living CD2^+^ CD8β^+^ HELIOS^+^ cells among T cell subsets is shown for one representative donor and **(D)** for 51 donors. **(E)** The prevalence of HELIOS^+^ cells in living CD2^+^ CD8β^+^ cells is compared between healthy donors and COVID-19 patients and **(F)** between blood and tumors from ovarian cancer patients. The average age of cancer patients was 61 years old, while the average was 55 for the blood donors. These averages are not different according to an unpaired t-test. **(G)** Frequency of Ki-67^+^ cells and **(H)** CD38^+^ cells among living CD2^+^ CD8β^+^ cells are shown in healthy donors and COVID-19 patients. **(I)** Percentage of Ki-67-positive cells in HELIOS^+^ and HELIOS^-^ CD8 TILs from ovarian cancer samples. **(J)** Representative donors’ data showing the TOX expression in non-naïve CD8 T-cells from blood or tumor, and gating strategy to identify TOX^hi^ cells. **(K)** Percentage of TOX^hi^ cells in HELIOS- or HELIOS+ non-naive CD8 T-cell from tumor samples. Data from 23 donors. Mean +/- SEM are shown. P values * = < 0,05, ** = < 0,01, *** = < 0,001 (one-way ANOVA).

To determine whether HELIOS^+^ CD8 T cells are naïve or memory, we analyzed peripheral blood mononuclear cells (PBMCs) using flow cytometry. HELIOS^+^ CD8 T cells were present in all 58 donors with a mean of 21% of CD8 T cells (**Figures 1A, B**). This observation is consistent with a previous study, which reported a mean of 15% of CD8 T cells in 5 donors (15). Furthermore, co-staining CCR7 and CD45RA revealed that HELIOS is expressed in some naïve CD8 T cells, but that most HELIOS^+^ CD8 T cells have encountered their antigen and belong mostly to the T_EM_ (CCR7^-^/CD45RA^-^) or T_EMRA_ (CCR7^-^/CD45RA^+^) subsets (**Figures 1C, D**) (23–26).

We also detected the presence of HELIOS^+^ CD8 T cells in the blood of COVID-19 patients, as well as in the blood and tumors of ovarian cancer patients (**Figures 1E, F**). The frequency of HELIOS^+^ CD8 T cells was found to be comparable between healthy donors and COVID-19 patients (**Figure 1E**). In ovarian tumors, the frequency of HELIOS^+^ CD8 T cells was slightly lower compared to that in the blood of ovarian cancer patients (**Figure 1F**).

In blood from hospitalized COVID-19 patients, a similar proportion of activated HELIOS^+^ CD8 T cells and HELIOS^-^ counterparts were detected as indicated by positive staining for Ki-67 and CD38 (**Figures 1G, H**). This indicates that activated HELIOS^+^ CD8 T cells can be found in response to a viral infection, specifically in the case of SARS-CoV2 infection. Similar results were observed in ovarian tumors, where Ki-67 staining also indicated the activation of HELIOS^+^ CD8 T cells (**Figure 1I**). Interestingly, a larger fraction of HELIOS^+^ CD8 T cells in ovarian tumors exhibited high expression of exhaustion marker TOX compared to their HELIOS^-^ counterparts (**Figures 1J, K**) (3, 27, 28).

Next, we evaluated the functionality of HELIOS^+^ CD8 T cells in comparison to their HELIOS^-^ counterparts in the blood of healthy donors.

### HELIOS^+^ CD8 T cells produce low levels of IFN-γ and TNF-α

To assess intracellular IFN-γ production by CD8 T cells, the cells were activated with plate-bound anti-CD3 antibodies for 5h, followed by flow cytometry analysis. As shown in **Figure S1**, the majority of IFN-γ-producing CD8 T cells express high levels of T-BET (29). Therefore, our IFN-γ production analysis focused on these T-BET high-expressing CD8 T cells. Moreover, considering that the majority of HELIOS^+^ CD8 T cells belong to the T_EM_ and T_EMRA_ subsets, our analysis specifically targeted these 2 subpopulations.

**Figure 2:**
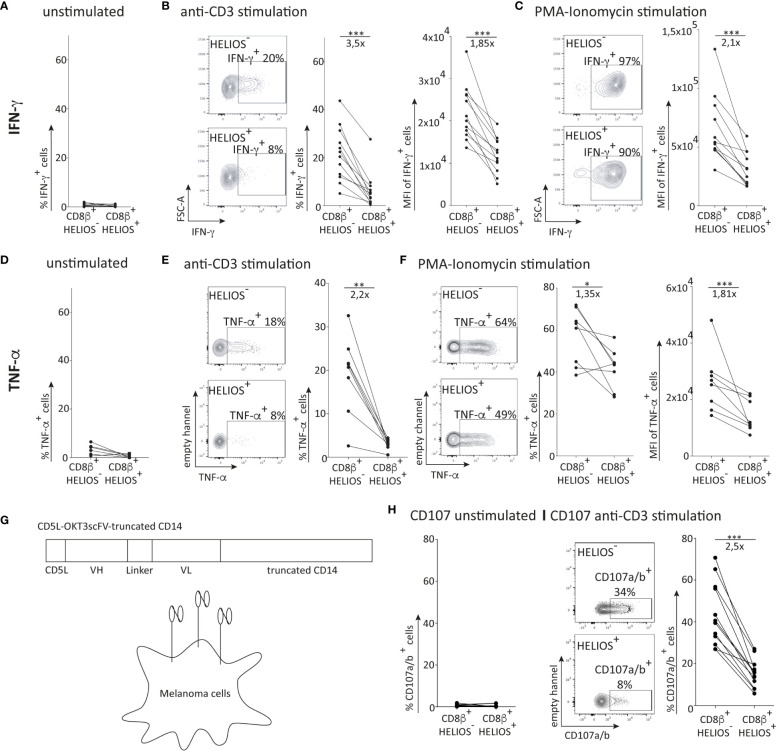
Association of HELIOS expression in CD8 T cells with impaired effector functions. PBMCs were thawed for 2h at 37°C with DNase. The cells were then stimulated with coated anti-CD3 (1μg/ml) or PMA (1ng/ml) and ionomycin (1μg/ml) for 5h at 37°C., Brefeldin A was added after the first hour of stimulation to block cytokine secretion. At the end of the 5h stimulation, cells were stained for viability, CD2, CD8β, CCR7 and CD45RA. Subsequently, the cells were fixed and permeabilized overnight, followed by intracellular staining for T-BET, HELIOS and IFN-γ. The samples were analyzed by flow cytometry. **(A)** Percentages of IFN-γ^+^ cells for resting T cells are shown **(B)** Representative plots for anti-CD3 activation are shown for T_EM_ for one donor and the percentages of IFN-γ^+^ cells and median fluorescence intensity are shown for T_EM_ of 12 donors. **(C)** Representative plots for PMA-ionomycin activation are shown for T_EM_ for one donor and the median fluorescence intensity is shown for T_EM_ of 12 donors. **(D)** Percentages of TNF-α^+^ cells for resting T cells are shown. **(E)** Representative plots for anti-CD3 activation are shown for T_EM_ for one donor and the percentages of TNF-α^+^ cells are shown for T_EM_ of 8 donors. **(F)** Representative plots for PMA-ionomycin activation are shown for T_EM_ for one donor and the percentage of TNF-α^+^ cells and median fluorescence intensity are shown for T_EM_ of 8 donors. **(G)** Representation of the membrane-bound anti-CD3 structure (inspired from (Leitner et al., 2010)). **(H)** Percentages of CD107a/b^+^ cells for resting T cells are shown. **(I)** Representative plots for membrane-bound anti-CD3 activation are shown for T_EM_ for one donor and percentage of CD107a/b^+^ cells are shown for T_EM_ of 12 donors. Mean +/- SEM are shown. P values *** = < 0,01, *** = < 0,001 (paired t test).

After 5h of activation, HELIOS^+^ CD8 T_EM_ and T_EMRA_ produce less IFN-γ than HELIOS^-^ cells both in terms of frequency and intensity (**Figures 2A, B**; **Figures S2A, B, E**). Even with a potent stimulus such as PMA-ionomycin, which bypasses early TCR signalling events, HELIOS^+^ CD8 T_EM_ and T_EMRA_ produce less IFN-γ than HELIOS^-^ CD8 T cells (F**igure 2C**; **Figures S2C-E**).

Similar trends were observed for TNF-α secretion. HELIOS^+^ CD8 T_EM_ and T_EMRA_ secreted less TNF-α than HELIOS^-^ cells (**Figures 2D–F**). These results collectively indicate the presence of T-BET^high^ CD8 T cells with a limited capacity for IFN-γ production and TNF-α secretion, characterized by the expression of HELIOS.

### HELIOS^+^ CD8 T cells exhibit reduced degranulation capacity

We also assessed the degranulation ability of HELIOS^+^ CD8 T cells. CD8 T cells were activated with melanoma cells expressing a membrane-bound form of anti-CD3 (**Figure 2G**). CD107a/b were labeled to measure T cell degranulation after 5h. The data clearly shows a degranulation defect in HELIOS^+^ CD8 T cells compared to HELIOS^-^ counterparts (**Figures 2H, I**), suggesting a reduced killing capacity of HELIOS^+^ CD8 T cells.

### HELIOS^+^ CD8 T cells are heterogeneous

To gain a comprehensive understanding of the characteristics of HELIOS^+^ CD8 T cells, we compared them to HELIOS^-^ CD8 T cells using RNA-sequencing. CD8 blood lymphocytes from 6 donors were either activated for 5h or left non-activated. Activation with plate-bound anti-CD3 was used to detect cytokine production capacities. After activation and intracellular staining for HELIOS, cells were sorted by flow cytometry into HELIOS^+^ and HELIOS^-^ populations (**Figure S3A**). However, the fixation and permeabilization steps required for intracellular HELIOS staining lead to RNA degradation. To prevent this, Rnase inhibitors were added to the buffers until RNA extraction was completed, as previously described (30) (**Figure S4**).

**Figure 3:**
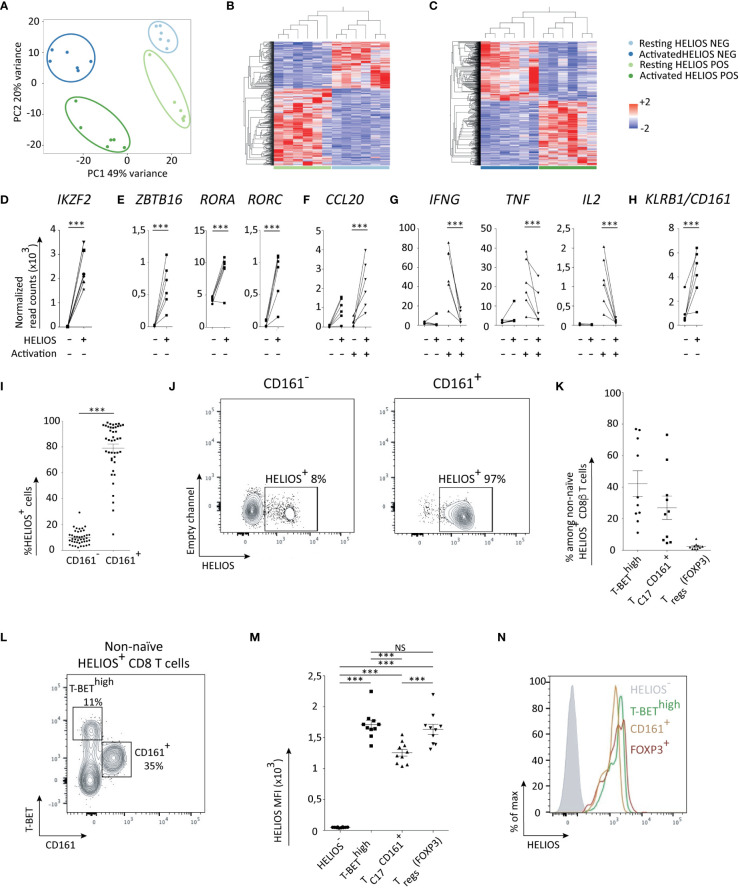
HELIOS^+^ CD8 T cells consist of at least two subpopulations: T_C17_ and T-BET^high^ CD8 T cells. **(A–I)** Frozen MACS-sorted CD8 PBLs from 6 donors were thawed for 2h at 37°C and then activated with plate-bound anti-CD3 antibody (1μg/ml) for 5h at 37°C. Cells were labeled for viability, CD2, CD8β, CCR7 and CD45RA. Then, cells were fixed and permeabilized overnight and stained intracellularly for HELIOS in presence of RNase inhibitors at 4°C. HELIOS^+^ and HELIOS^-^ CD8 T_EM_ (CCR7^-^ CD45RA^-^) were sorted by flow cytometry. RNA was then extracted to perform paired-end RNA sequencing. Data normalization and analysis were done with DESeq2. **(A)** The principal component analysis is shown. **(B)** Heatmaps for differentially expressed genes of non-activated and **(C)** activated samples are shown with hierarchical clustering on genes and samples. Expression values of selected transcription factors for unstimulated samples are shown for **(D)** HELIOS, **(E)** ZBTB16, RORC and RORA. Expression values for selected cytokines **(F)** upregulated in HELIOS^+^ CD8 T_EM_ and **(G)** downregulated ones are shown. **(H)** Expression values for unstimulated samples are shown for the surface marker KLRB1/CD161. P values * = < 0,05, ** = < 0,01, *** = < 0,001 (DESeq 2 paired samples analysis). **(I–N)** PBMCs were thawed for 2h at 37°C. Cells were labeled for viability, CD2, CD8β, CCR7, CD45RA and CD161. Then, cells were fixed and permeabilized overnight and stained intracellularly for HELIOS. Samples were analyzed by flow cytometry. **(I)** Percentages of HELIOS^+^ cells among CD161^-^ and CD161^+^ CD8 T_EM_ (CCR7^-^ CD45RA^-^) are shown for 42 donors and **(J)** one donor. **(K)** Frequency of T_C1_ (T-BET^high^), T_C17_ (CD161^+^) and FOXP3^+^ cells among HELIOS^+^ CD8 T cells are shown for 10 donors and **(L)** for one donor. **(M)** The intensity (MFI) of HELIOS staining is shown for one donor and **(N)** for 10 donors. Mean +/- SEM are shown. P values *** = < 0,001 (**(I)** Wilcoxon signed-rank test and **(M)** one-way ANOVA).

Principal component analysis (PCA) plot clearly demonstrates the distinct nature of HELIOS^+^ and HELIOS^-^ CD8 T cells subpopulations (**Figure 3A**). A total of 1194 genes and 2605 genes were found differentially expressed before and after activation respectively (**Figures 3B, C**).

As expected, the expression of *IKZF2*, the gene encoding HELIOS, matched with the sorted populations (**Figure 3D**). Interestingly, genes coding T_H17_/T_C17_ transcription factors *RORC*, *RORA* and *ZBTB16* were more expressed in HELIOS^+^ CD8 T cells (**Figure 3E**). The CD8 T cells that express RORγt, the T_H17_ transcription factor, are usually referred to as T_C17_ (31–34). Our findings thus suggest the presence of T_C17_ cells within the HELIOS^+^ CD8 T cell population.

T_C17_ cells involved in defense against extracellular pathogens such as *Candida Albicans* and *Staphylococcus* (35). In our experiments, HELIOS^+^ CD8 T cells express less *IFNG*, *TNF* and *IL2* but express more *CCL20*, a chemokine associated with T_C17_/T_H17_ cells (**Figures 3F, G**). Additionally, HELIOS^+^ CD8 T cells demonstrated higher expression of *KLRB1* (CD161), the surface marker for T_C17_/T_H17_. This was confirmed by flow cytometry (**Figures 3H–J**) (34).

CD161 is often used as a surface marker for the identification and characterization of T_C17_ cells (34). By co-staining CD161, T-BET and FOXP3, we propose that HELIOS^+^ CD8 T cells comprises a minimum of two distinct subpopulations: T_C17_ (CD161^+^) and T-BET^high^ CD8 T cells (**Figures 3K, L**). The HELIOS^+^ CD161^+^ T_C17_ subpopulation appears to express slightly lower levels of HELIOS compared to the HELIOS^+^ T-BET^high^ CD8 T cells (**Figures 3M, N**).

### Deciphering the heterogeneity of HELIOS^+^ CD8 T cells

Given the observed heterogeneity of HELIOS^+^ CD8 T cells, we conducted RNA-sequencing on five CD8 T cell subpopulations: HELIOS^+^ CD161^+^ cells, HELIOS^+^ T-BET^low/int^ and T-BET^high^ cells, and also HELIOS^-^ T-BET^low/int^ and T-BET^high^ cells (**Figure S3B**). The main goal pursued was to obtain a picture of the panel of differentially expressed transcription factors and cytokines of each subpopulation. The second one was to obtain a combination of surface markers to isolate pure living HELIOS^+^ T-BET^high^ CD8 T cells.

**Figure 4:**
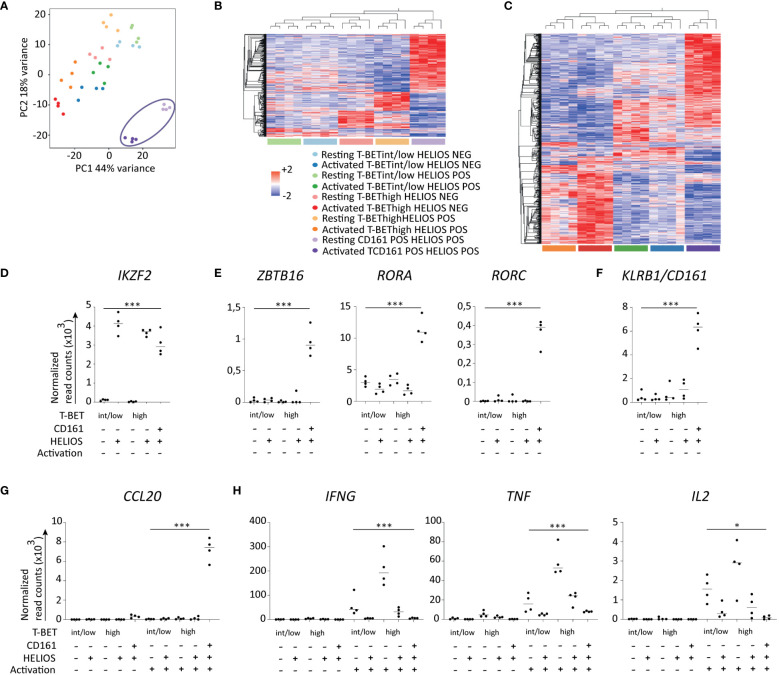
Transcriptomes of HELIOS^+^ and HELIOS^-^ CD8 T cell subpopulations. Frozen MACS-sorted CD8 PBLs from 4 donors were thawed for 2h at 37°C and then activated with plate-bound anti-CD3 antibody (1μg/ml) for 5h at 37°C. Cells were labeled for viability, CD8β, CCR7, CD45RA and CD161. Then, cells were fixed and permeabilized overnight and stained intracellularly for HELIOS and T-BET in presence of RNase inhibitors at 4°C. HELIOS^+^ and HELIOS^-^ T-BET^high^, T-BET^int/low^ and CD161^+^ T_EM/EMRA_ CD8 T cells (CCR7^-^) were sorted by flow cytometry. RNA was then extracted to perform paired-end RNA sequencing. Data normalization and analysis were done with DESeq2. **(A)** The principal component analysis is shown. **(B)** Heatmaps for differentially expressed genes of resting and **(C)** activated samples are shown with hierarchical clustering on genes and samples. Expression values of selected transcription factors for resting samples are shown for **(D)** HELIOS, **(E)** ZBTB16, RORC and RORA and **(F)** KLRB1/CD161. Expression values for selected cytokines **(G)** upregulated in HELIOS^+^ CD8 T_EM_ and **(H)** downregulated ones are shown. P values * = < 0,05, *** = < 0,001 (Qlucore ANOVA analysis).

Principal component analysis indicates that CD161^+^ HELIOS^+^ CD8 T cells represent a clearly distinct population (**Figure 4A**). The differences among the remaining populations are more subtle although they can still be observed, as depicted in the grouped hierarchical clustering (**Figures 4B, C**). A total of 1463 genes and 3011 genes were found to be differentially expressed before and after activation, respectively.

As anticipated, the expression of *IKZF2*, which encodes HELIOS, aligns with the subpopulations (**Figure 4D**). Genes coding transcription factors associated with T_C17_, namely *ZBTB16*, *RORC* and *RORA*, and surface marker, *CD161*/*KLRB1*, show higher expression by CD161^+^ HELIOS^+^ CD8 T cells (**Figures 4E, F**). Regarding cytokines, *CCL20* is expressed at higher levels in the CD161^+^ HELIOS^+^ CD8 T cells subpopulation as expected (**Figure 4G**). On the other hand, *IFNG* and *TNF* are more expressed by HELIOS^-^ T-BET^high^ CD8 T cells. However, no statistically significant differences were found for *IL2* (**Figure 4H**).

Besides analysing the HELIOS^+^ CD8 T cell subpopulation, the second aim of the experiment was to identify surface markers that could effectively isolate viable HELIOS^+^ TBET^high^ CD8 T cells. Unfortunately, we were unable to identify a reliable combination of surface markers that could be validated using flow cytometry.

### HLA-E-restricted CD8 T cells: an intriguing CD8 T cell population partially expressing HELIOS

**Figure 5:**
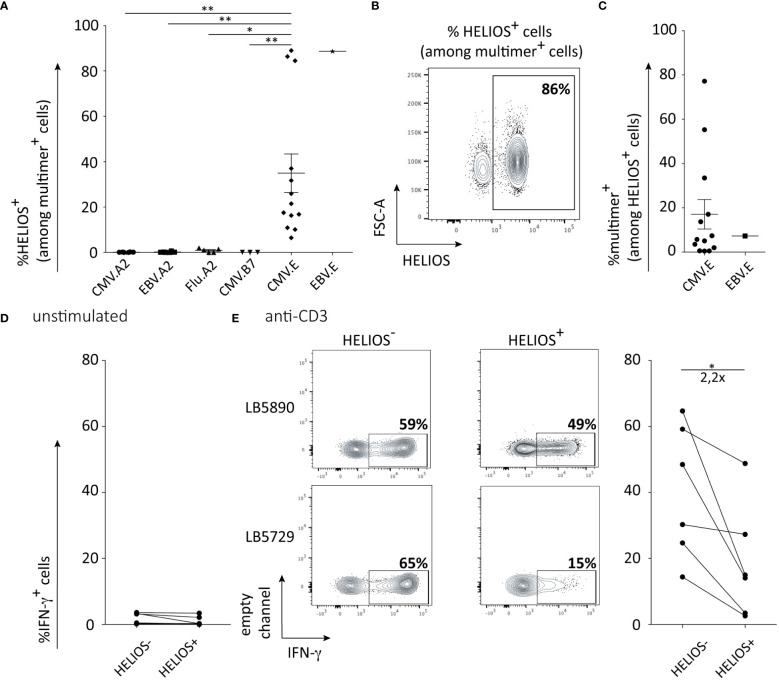
HELIOS^+^ CD8 T cells are found in HLA-E-restricted T cells. PBMCs were thawed and rested for 2h at 37°C. The cells were labeled with a multimer and stained for viability, CD2, CD8β, CCR7, CD45RA. Next, cells were fixed and permeabilized overnight and stained intracellularly for HELIOS at 4°C. Samples were analyzed by flow cytometry. **(A)** Frequency of HELIOS^+^ cells in living CD2^+^ CD8β^+^ multimer^+^ cells are shown for virus-derived peptides presented by HLA-A2, HLA-B7 and HLA-E. CMV.A2 (pp65: NLVPMVATV) and EBV.A2 (BMF-L1: GLCTLVAML) multimer^+^ CD8 T cells were detected in 6 out of 11 HLA-A2 donors and 5 out of 11 for Flu.A2 (M1: GILGFVFTL) multimer. CMV.B7 (pp65: RPHERNGFTVL or TPRVTGGGAM) multimer+ CD8 T cells were detected in 2 out 4 HLA-B7 donors for peptide RPHERNGFVTL and 1 out of 4 HLA-B7 donors for peptide TPRVTGGGAM. CMV.E (UL40: VMAPRTLIL) multimer^+^ CD8 T cells were detected in 13 out of 20 non-typed donors. EBV.E (BZF-L1: SQAPLPCVL) multimer^+^ CD8 T cells were detected in 1 out of 20 non-typed donors. **(B)** Percentages of HELIOS^+^ cells among multimer^+^ cells are shown for one donor with the CMV.E multimer. **(C)** Percentages of multimer^+^ cells among HELIOS^+^ CD8 T cells are shown for CMV.E and EBV.E multimers. **(D)** Frequency of IFN-γ^+^ cells among multimer^+^ cells is shown at resting state for 6 donors. **(E)** Frequency of IFN-γ^+^ cells among multimer^+^ cells is shown after 5h anti-CD3 stimulation for 6 donors and representative FACS plots are shown for 2 donors. Mean +/- SEM are shown. P values * = < 0,05, ** = < 0,01 (Kruskal-Wallis test).

As HELIOS seems to mainly be expressed by memory CD8 T cells, we were wondering if they participate in the immune response against viral antigens. We evaluated HELIOS expression in viral-specific CD8 T cells targeting various epitopes presented by HLA-A2 or HLA-B7. The absence of HELIOS-expressing cells in CD8 T cells targeting CMV, EBV and influenza epitopes presented by HLA-A2 is quite striking (**Figure 5A**). This finding was reproduced using a CMV epitope presented by HLA-B7 (**Figure 5A**). We next looked at viral peptides presented by HLA-E for CMV and EBV. HELIOS-expressing cells were found in CD8 T cells recognizing a CMV epitope presented by HLA-E (**Figures 5A–C**). This observation was also reproduced on 1 donor for an EBV epitope presented by HLA-E (**Figure 5A**). We tried to confirm these results for peptides derived from mycobacterium tuberculosis and presented by HLA-A2 or HLA-E but we failed to detect CD8 T cells against these HLA-peptide complexes in tuberculosis patient as previously described (36).

**Figure 6:**
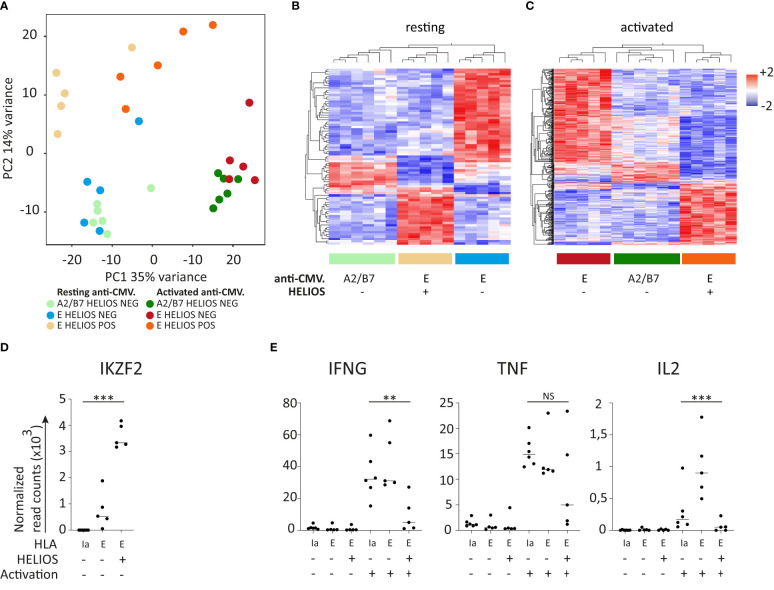
HELIOS^+^ anti-CMV.E CD8 T cells are distinct from their HELIOS^-^ anti-CMV counterparts and express low levels of IFN-γ. Frozen MACS-sorted CD8 PBLs from 6 donors were thawed for 2h at 37°C and then activated with plate-bound anti-CD3 antibody (1μg/ml) for 5h at 37°C. Cells were labeled for viability, CD8β, CCR7, CD45RA and CMV tetramer. Then, cells were fixed and permeabilized overnight and stained intracellularly for HELIOS in presence of RNase inhibitors at 4°C. HELIOS^+^ and HELIOS^-^ anti-CMV.A2/B7/2 CD8 T cells were sorted by flow cytometry. RNA was then extracted to perform paired-end RNA sequencing. Data normalization and analysis were done with DESeq2. **(A)** The principal component analysis is shown. **(B)** Heatmaps for differentially expressed genes of resting and **(C)** activated samples are shown with hierarchical clustering on genes and samples. **(D)** Expression values for resting samples are shown for HELIOS. **(E)** Expression values for selected cytokines are shown. P values * = < 0,05, ** = < 0,01, *** = < 0,001 (Qlucore ANOVA analysis).

Next, we evaluated the ability of these HLA-E-restricted HELIOS^+^ CD8 T cells to produce intracellular IFN-γ. Similar to the results obtained when comparing the entire HELIOS^+^ T-BET^high^ CD8 T cell population with the HELIOS^-^ T-BET^high^ CD8 T cell population, anti-CMV.E HELIOS^+^ T-BET^high^ CD8 T cells produced less IFN-γ than anti-CMV.E HELIOS^-^ T-BET^high^ CD8 T cells (**Figures 5D, E**). We conducted RNA-sequencing on HELIOS^-^ anti-CMV.A2/B7 and on HELIOS^-^ and HELIOS^+^ anti-CMV.E CD8 T cells (**Figure S3C**). Resting and 5h activated samples were sorted as described in previous sections. The PCA plot demonstrates that HELIOS^+^ and HELIOS^-^ CD8 T cells are quite distinct from each other (**Figure 6A**). A total of 78 genes and 554 genes were found to be differentially expressed before and after activation respectively (**Figures 6B, C**). Only a small number of genes were differentially expressed between these 3 populations before activation. However, upon activation, more differentially expressed genes were detected.

As expected, IKZF2 was expressed at higher levels in HELIOS^+^ anti-CMV.E CD8 T cells, but interestingly, a background signal was detectable in HELIOS^-^ anti-CMV.E CD8 T cells and not in HELIOS^-^ anti-CMV.A2/B7 CD8 T cells (**Figure 6D**). *IFNG* was more highly expressed by HELIOS^-^ anti-CMV.A2/B7/E CD8 T cells, confirming the flow cytometry data. The same observation holds true for *IL2* but not for *TNF* (**Figure 6E**).

## Discussion

Here, we report that HELIOS is frequently expressed by human CD8 T cells obtained from blood donors, with frequencies reaching up to 60% of these cells. HELIOS is found in diverse CD8 T cell populations, including T-BET^high^ cells that produce low cytokine levels and T_C17_ cells.

It is important to consider the origin of HELIOS expression: whether it is acquired in the thymus or in the periphery. The majority of HELIOS^+^ CD8 T cells are T_EM_ and T_EMRA_, indicating that they are antigen-experienced T cells. However, few HELIOS^+^ CD8 T cells express CCR7 and CD45RA, markers associated with naïve T cells. This could suggest that HELIOS expression might be acquired during T cell maturation in the thymus and is not induced during or after CD8 T cell priming in the periphery. Similarly, in mice, HELIOS is exclusively expressed by thymic-derived CD4 T_regs_ and not by peripherally-induced CD4 T_regs_ (9).

Our findings suggest that a subset of HELIOS^+^ CD8 T cells are HLA-E-restricted (4). In a murine model of experimental autoimmune encephalitis (EAE), CD8 Tregs recognize and eliminate pathogenic CD4 T cells. EAE development is mediated by anti-MOG (Myelin Oligodendrocyte Glycoprotein) TCR Vβ8.2 CD4 T cells. Immunizing mice with a non-self-antigen, which triggers a TCR Vβ8.2 CD4 T cell response, protects mice from developing EAE. This protection is mediated by Qa-1-restricted CD8 Tregs that recognize peptides from Vβ leader sequences presented by Qa-1 (murine homolog of HLA-E) and lyse them (37–41). CD8 Tregs are also able to inhibit lupus-like autoimmune disease through the specific recognition of Qa-1/peptide complexes on follicular helper CD4 T cells. HELIOS-deficient CD8 T_regs_ failed to protect mice against autoimmune disease, highlighting a potential shared role for HELIOS in CD8 T_regs_ and CD4 T_regs_ (4).

This raises the question of whether there is a connection between HLA-E restriction and HELIOS expression? If such a link exists, then why is HELIOS expressed by a subset of HLA-E-restricted CD8 T cells? Interestingly, in mice, CD8 T cells restricted by the MHC class Ib Qa-1 (HLA-E homolog) undergo positive selection in the thymus by different cell types compared to MHC class Ia-restricted CD8 T cells. While MHC class Ia-restricted CD8 T cells undergo positive selection on thymic epithelial cells, Qa-1-restricted CD8 T cells do so on either thymic epithelial cells or hematopoietic cells (42, 43). Consequently, MHC class Ib-restricted CD8 T cells receive distinct signals during positive selection. If some HLA-E-restricted CD8 T cells are positively selected by hematopoietic cells (such as dendritic cells), this could potentially explain why only a fraction of HLA-E-restricted CD8 T cells express HELIOS. Moreover, a recent study has described an unconventional CD8 T cell population expressing HELIOS for which the differentiation pathway in the thymus differs from conventional T cell lineage (44). However, HELIOS expression can be induced in the periphery in the presence of persistent antigen. Studies in mice have demonstrated that *HELIOS* mRNA is upregulated in exhausted CD8 T cells compared to naïve, effector or memory CD8 T cells (1, 3, 6). Given our current understanding of HELIOS and human CD8 T cells, it is likely that some HELIOS-expressing CD8 T cells acquire HELIOS expression in the thymus during T cell maturation. However, under specific circumstances or certain immune contexts HELIOS expression is likely acquired in the periphery.

In our study we showed that HELIOS^+^ CD8 T cells are present in ovarian tumors and both HELIOS^+^ and HELIOS^-^ CD8 T cells exhibited high expression of exhaustion marker TOX. It has been well-established in mice that only anti-tumor CD8 TILs express TOX, while bystander TILs do not (28). This leads to a tentative conclusion – the existence of anti-tumor CD8 T cells expressing HELIOS – and raises three hypotheses (1): the existence of HLA-E-restricted anti-tumor CD8 T cells since we demonstrated that only HLA-E-restricted CD8 T cells express HELIOS (2); HELIOS expression in some exhausted TOX^hi^ CD8 TILs independent of HLA-E restriction (3); a combination of both possibilities. While TOX is transiently expressed in non-exhausted CD8 T cells after activation, TOX expression in our tumor samples correlated with poor IFN-γ production and therefore exhaustion (data not shown).

We established a correlation between HELIOS expression and low expression of IFN-γ, TNF-α and IL-2, but it remains unclear if HELIOS is the direct cause of this effect. Existing research in mice has shown that HELIOS binds to the *IL2* promoter in CD4 T_regs_ in mice and repress its expression (45). Thus, a similar mechanism could be operating in CD8 T cells. Moreover, the presence of core sequence motifs (GGGA and GGAAA) in the *IFNG* promoter further supports the likelihood of its binding to the promoter region of IFN-γ (46).

While confirmatory data are lacking, the lower IFN-γ production upon PMA-ionomycin stimulation indicates that late TCR signalling events or even *IFNG* gene accessibility differ between HELIOS^+^ and HELIOS^-^ CD8 T cells. Altogether it is likely that HELIOS repress the transcription of effector cytokines including IL-2 and IFN-γ.

We demonstrated that HELIOS^+^ CD8 T cellsare heterogeneous and contain several subpopulations including T_C17_ and T-BET^high^ CD8 T cells with low effector functions. This enlightens at least partially the potential role of HELIOS^+^ CD8 T cells. T_C17_ help control fungal and viral infections (47–49). *In vitro* polarized T_C17_ protect mice against lethal influenza infection comparably to *in vitro* polarized T_C1_ (47). MHC class Ib-restricted T_C17_ also maintain skin homeostasis by controlling skin-specific microbiota (50, 51). T_C17_ cells are also involved in inflammatory diseases such as psoriasis; their accumulation is observed in skin psoriatic lesions (52). However, the function of T-BET^high^ HELIOS^+^ CD8 T cells with diminished effector functions remains unclear. It is puzzling to maintain such a significant quantity of CD8 T cells with limited functional potential within our bloodstream.

To conclude, our findings indicate that HELIOS should not be solely considered as a transcription factor for T_regs_ or exhausted CD8 T cells.

## Data availability statement

The datasets presented in this study can be found in online repositories. The names of the repository/repositories and accession number(s) can be found below: GSE243096 (GEO).

## Ethics statement

The studies involving humans were approved by Cliniques Universitaires Saint-Luc (CUSL) ethical committee: (2017/11OCT/478 – Belgian n°: B403201734113, 2020/27AVR/246 and 2022/14NOV/423 – Belgian n°: BE4032022000129). The studies were conducted in accordance with the local legislation and institutional requirements. The participants provided their written informed consent to participate in this study.

## Author contributions

DN: Writing – original draft, Writing – review & editing, Conceptualization, Data curation, Formal analysis, Investigation, Methodology, Software. TH: Writing – review & editing, Conceptualization, Data curation, Formal analysis, Investigation, Methodology, Software. AA: Writing – review & editing. ND: Writing – review & editing. CV: Writing – review & editing. AB: Writing – review & editing. CW: Writing – review & editing. ML: Writing – review & editing. J-LS: Writing – review & editing. QD: Writing – review & editing. CD: Writing – review & editing. AH: Writing – review & editing. VM: Writing – review & editing. MD: Writing – review & editing. PB: Conceptualization, Writing – review & editing, Funding acquisition, Project administration, Resources, Supervision.
