# Supplementary material for: HELIOS-expressing human CD8 T cells exhibit limited effector functions
Source: Front Immunol. 2023 Dec 22;14:1308539. doi: 10.3389/fimmu.2023.1308539 (PMC10770868; doi:10.3389/fimmu.2023.1308539)
Supplement: Supplementary file 1 [file DataSheet_1.pdf]

SUPPLEMENTARY FIGURES

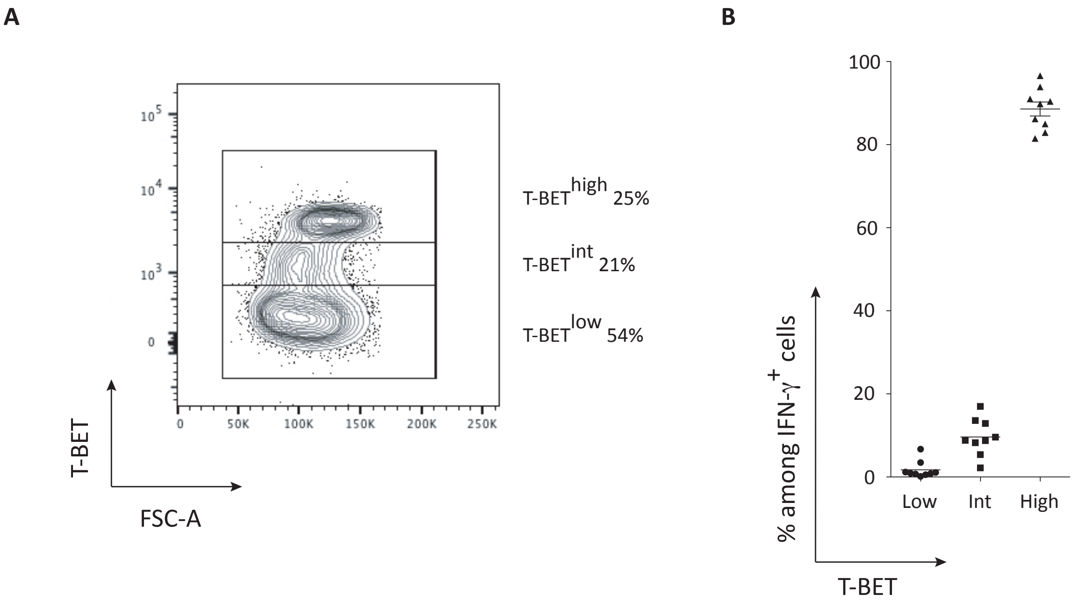

Supplementary Figure 1. The majority of IFN-γ⁺ **CD8 T cells express high levels of T-BET.** *PBMCs were thawed for 2h at 37°C with 5U/ml DNase. Cells were stimulated with coated anti-CD3 (1μg/ml) for 5h at 37°C. After the first hour of stimulation, 5μg/ml of Brefeldin A was added to block cytokine secretion. At the end of the 5h stimulation cells were stained for viability, CD2, CD8β, CCR7 and CD45RA. Cells were then fixed and permeabilized overnight and stained intracellularly for T-BET, HELIOS and IFN-γ. Samples were analyzed by flow cytometry.* ***(A)*** *Representative plots for T-BET staining in CD8 T cells.* ***(B)*** *Percentage of IFN-γ^{+} cells and shown for T-BET^{high}, T-BET^{int} and T-BET^{low} CD8 T cells of 9 donors.*

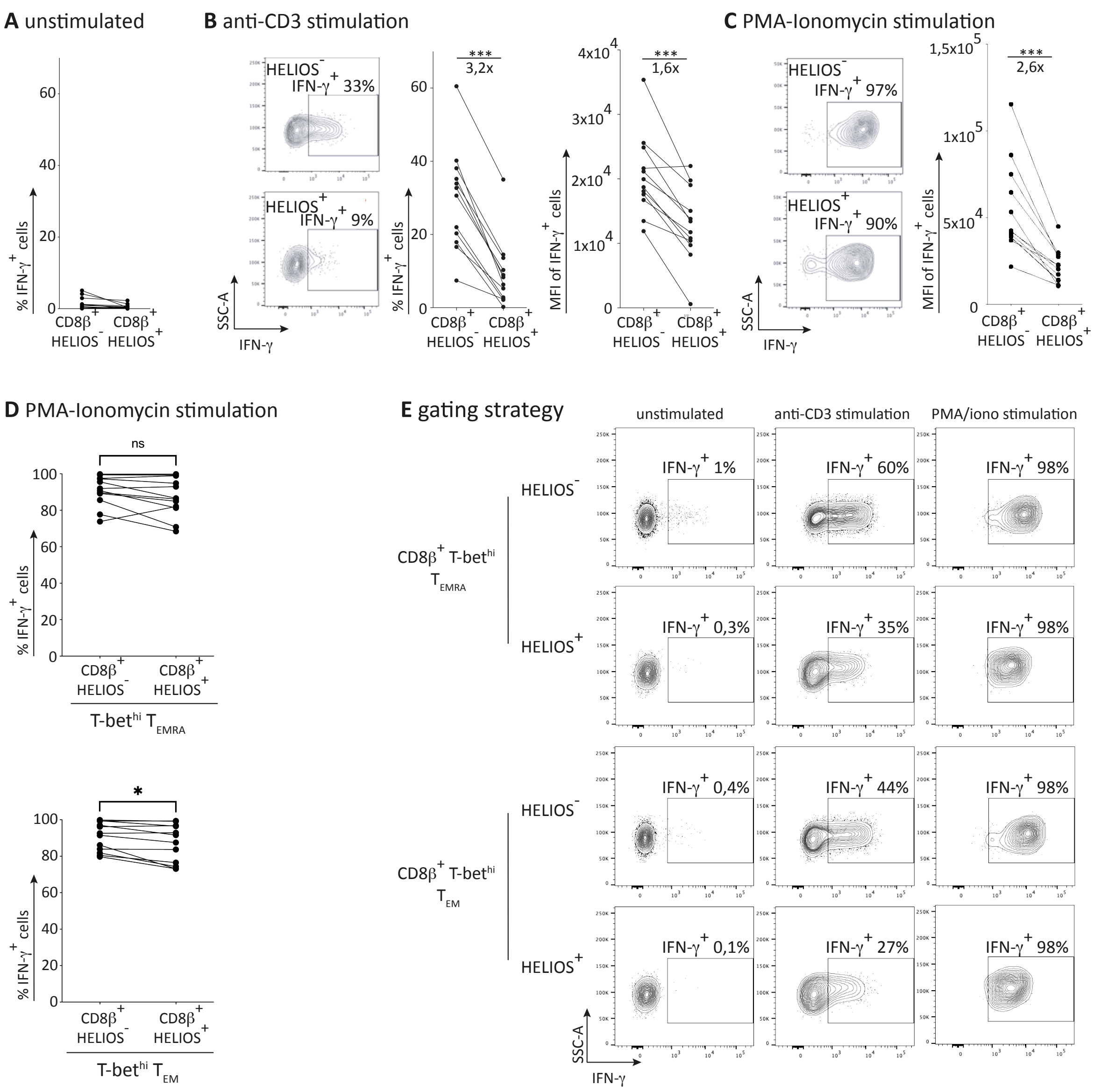

Supplementary Figure 2. HELIOS⁺ T-BET^{high} CD8 T_{EMRA} cells produce less IFN-γ than HELIOS⁻ CD8⁺ T_{EMRA}. *PBMCs were thawed for 2h at 37°C with 5U/ml DNase. Cells were stimulated with coated anti-CD3 (1µg/ml) or PMA (1ng/ml) and ionomycin (1µg/ml) for 5h at 37°C. After the first hour of stimulation 5µg/ml of Brefeldin A was added to block cytokine secretion. At the end of the 5h stimulation cells were stained for viability, CD2, CD8β, CCR7 and CD45RA. Cells were then fixed and permeabilized overnight and stained intracellularly for T-BET, HELIOS and IFN-γ. Samples were analyzed by flow cytometry.* ***(A)*** *Percentages of IFN-γ$^+$ cells for resting T cells are shown.* ***(B)*** *Representative plots for anti-CD3 activation are shown for T_{EMRA} for one donor and percentage of IFN-γ$^+$ cells and median fluorescence intensity are shown for T_{EMRA} of 12 donors.* ***(C)*** *Representative plots for PMA-ionomycin activation are shown for T_{EMRA} for one donor and median of fluorescence intensity are shown for T_{EMRA} of 12 donors.* ***(D)*** *Percentages of IFN-γ$^+$ cells for PMA-ionomycin activated T_{EMRA} (upper panel) or T_{EM} (lower panel) of 12 donors. P values * = < 0,05, ** = < 0,01, *** = < 0,001 (paired t test).* ***(E)*** *Gating strategy to identify IFN-γ$^+$ cells upon stimulation with anti-CD3 or PMA-ionomycin.*

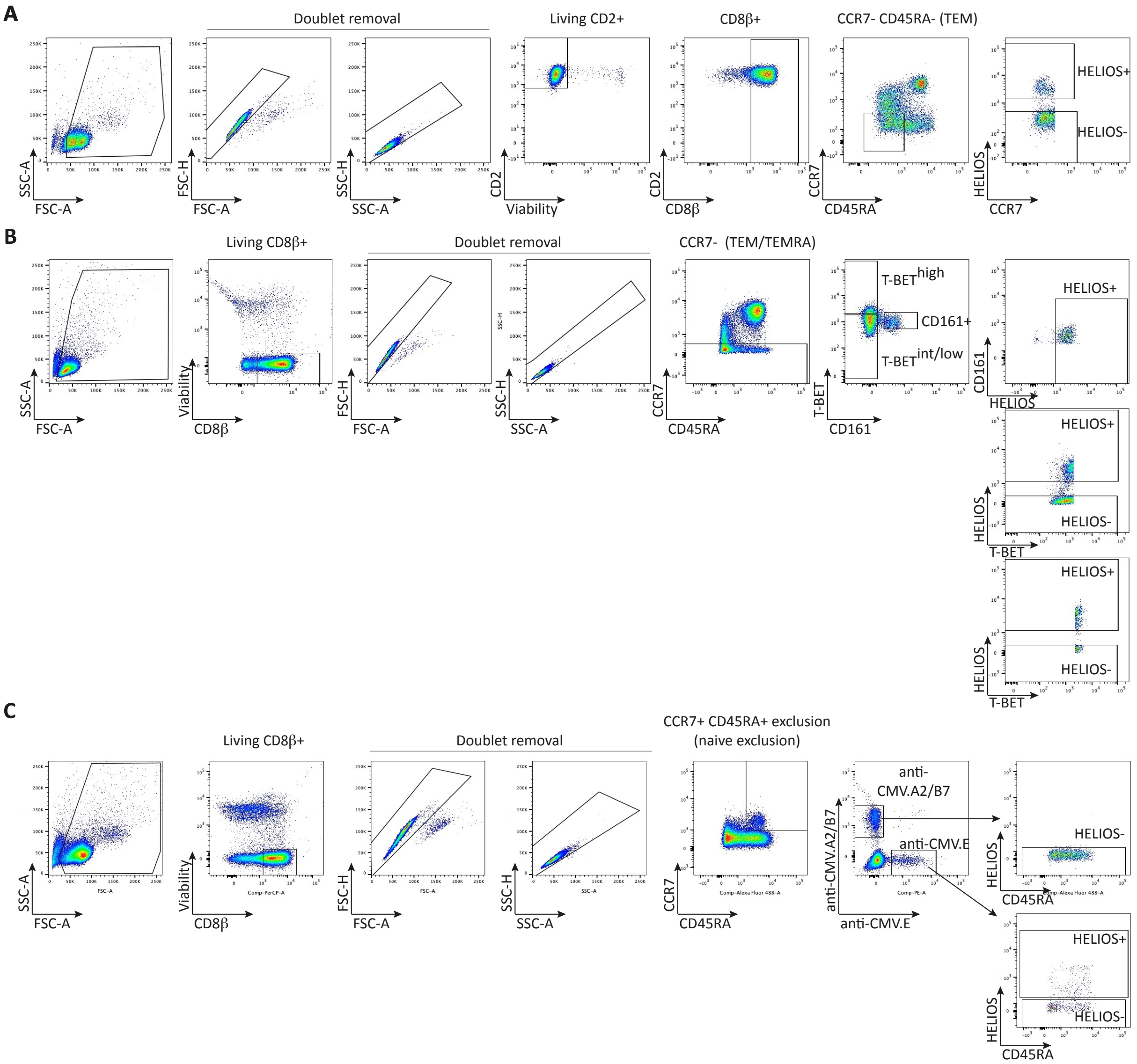

Supplementary Figure 3. Gating strategies used for FACS sorting. *MACS-sorted CD8 PBLs were thawed for 2h at 37°C in T cell medium with 5U/ml DNAse. Half of the cells were activated with plate bound anti-CD3 antibody (1μg/ml) for 5h at 37°C.* ***(A)*** *HELIOS$^+$ versus HELIOS$^-$ T$_{EM}$ sorting: cells were labeled at 4°C for viability, CD2, CD8β, CCR7 and CD45RA. Then, cells were fixed and permeabilized overnight and stained intracellularly for HELIOS in presence of RNase inhibitors at 4°C.* ***(B)*** *HELIOS$^+$ versus HELIOS$^-$ T-BET^{high}, T-BET$^{int/low}$ and CD161$^+$ sorting: cells were labeled at 4°C for viability, CD8β, CCR7, CD45RA and CD161. Then, cells were fixed and permeabilized overnight and stained intracellularly for HELIOS and T-BET in presence of RNase inhibitors at 4°C.* ***(C)*** *HELIOS$^+$ versus HELIOS$^-$ anti-CMV.A2/B7/E sorting: cells were labeled at 4°C for viability, CD8β, CCR7, CD45RA and tetramer. Then, cells were fixed and permeabilized overnight and stained intracellularly for HELIOS and T-BET in presence of RNase inhibitors at 4°C.* ***(A, B & C)*** *Cells were then sorted by flow cytometry and RNA was then extracted to perform paired-end RNA sequencing.*

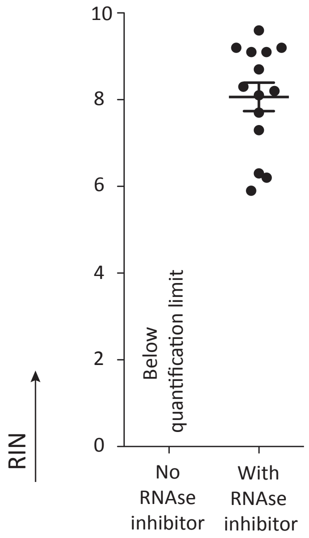

Supplementary Figure 4. RNA extraction from fixed/permeabilized cells requires RNAse inhibitors during intracellular staining. *PBMCs were thawed for 2h at 37°C with 5U/ml DNase. Cells were then submitted to extracellular and intracellular staining procedures. For the intracellular staining, cells were separated in two and RNAse inhibitors were added in half of them. Following RNA extraction, RNA integrity numbers were determined using bioanalyzer Agilent 2100 following the manufacturer protocol.*
