# Supplementary material for: HELIOS-expressing human CD8 T cells exhibit limited effector functions
Source: Front Immunol. 2023 Dec 22;14:1308539. doi: 10.3389/fimmu.2023.1308539 (PMC10770868; doi:10.3389/fimmu.2023.1308539)
Supplement: Supplementary file 2 [file DataSheet_2.pdf]

Supplementary Table 1: Description of the cohort of blood donors with hemochromatosis

**Hemochromatosis Donor**	**age at blood sampling**	**gender**	**% HELIOS+ in blood non naive CD8**
LB-1117	66	M	6.616
LB-5979	43	M	28
LB-3432	55	M	13.2
LB-5727	72	M	4.27
LB-5773	63	M	15.5
LB-554	56	M	23.7
LB-3340	62	M	16.8
LB-9183	25	M	13.7
LB-9181	71	M	8.89
LB-5949	67	M	28.4
LB-3329	58	M	12.3
LB-534	80	M	8.78
LB-670	75	M	21.5
LB-5920	26	F	25.4
LB-6022	44	M	26.3
LB-2050	78	F	28.2
LB-3300	55	F	14.5
LB-2369	65	F	17.5
LB-1758	67	M	13.5
LB-543	49	M	18.1
LB-6019	43	M	19.7
LB-6002	73	F	36.3
LB-5831	72	F	10.6
LB-9251	42	M	34.7
LB-3330	64	M	19.4
LB-2732	67	M	13.2
LB-3024	68	F	16.7
LB-9344	28	M	15.4
LB-9349	60	M	10.1
LB-9345	30	M	15.1
LB-5984	27	M	17.1
LB-9357	56	F	35.8
LB-9356	56	M	9.78
LB-2889	56	M	24.5
LB-9350	25	F	27.4
LB-9348	25	F	44
LB-9353	31	M	21.3
LB-9352	23	M	39
LB-9355	39	M	43.7
LB-9347	25	F	41.4
LB-2265	57	M	12.6
LB-2960	46	M	20.6
LB-3365	52	M	12.7
LB-3374	51	M	24.6
LB-9444	65	M	15.2
LB-5781	70	M	9.85
LB-5898	52	M	7.05
LB-9443	61	M	23.2
LB-9338	68	F	16
LB-3086	68	M	21
LB-5944	49	M	28.3
LB-3087	75	M	34.7
LB-5766	70	M	19.8
LB-9364	59	F	18.1
LB-3433	57	M	15.1
LB-2366	75	M	10.7
LB-9331	40	M	24.2
LB-9279	74	M	63.2

Supplementary Table 2: Description of the cohort of patients with ovarian cancer

Ovarian cancer patient	age of the patient	gender	Anatomical Pathology	FIGO staging	Diagnostic	neoadjuvant chemotherapy	% HELIOS+ in blood non naive CD8	% HELIOS+ in tumor non naive CD8 (TILs)	% TOX^{high} in HELIOS- non naive CD8 TILs	% TOX^{high} in HELIOS+ non naive CD8 TILs
LB-6151	75	F	endometrioid carinoma	IIA	Primodiagnostic	no	46.8	26.5	55.8	54.2
LB-9189	35	F	grade 3 serous carcinoma	IIIc	Primodiagnostic	no	32.7	26.3	53.2	53.2
LB-6119	44	F	grade 3 serous carcinoma	IIIc	Primodiagnostic	no	21.8	3.6	*na*	*na*
LB-9276	64	F	grade 3 serous carcinoma	IIIc	Primodiagnostic	no	17	4.94	7.53	26.5
LB-9263	75	F	grade 3 serous carcinoma	IIIB	Primodiagnostic	no	18.5	16.2	10.4	46.3
LB-9168	54	F	grade 3 serous carcinoma	IIIC	Primodiagnostic	no	12.5	18.3	29.3	41.1
LB-9280	70	F	grade 3 serous carcinoma	IIIC	Primodiagnostic	no	8.8	10.9	43.3	46.6
LB-9226	68	F	grade 2 serous carcinoma	IIIc	Primodiagnostic	yes	*na*	12.6	3.8	13.6
LB-9207	83	F	low grade serous carcinoma	IIIc	Primodiagnostic	no	10.3	5.2	*na*	*na*
LB-9256	72	F	grade 3 serous carcinoma	IIIc	Primodiagnostic	no	*na*	7.79	16.4	27.9
LB-9240	55	F	grade 3 serous carcinoma	Ivb	Primodiagnostic	yes	*na*	15.3	30.4	46.4
LB-9227	58	F	grade 3 serous carcinoma	IIIc	Primodiagnostic	no	36.4	11.6	35.7	40.1
LB-9257	49	F	grade 3 serous carcinoma	IIIC	Primodiagnostic	no	32.3	35.4	30.6	46
LB-9202	72	F	grade 3 serous carcinoma	IIIc	Primodiagnostic	yes	*na*	11.8	24.2	27.6
LB-6111	37	F	low grade serous carcinoma	IIIc	Primodiagnostic	yes	*na*	43.1	17.3	60.6
LB-9238	60	F	grade 3 serous carcinoma	IIIc	Primodiagnostic	no	*na*	9.33	41.2	33.3
LB-9216	83	F	grade 3 serous carcinoma	IIIb	Primodiagnostic	yes	*na*	10.3	5.01	15
LB-9148	56	F	grade 3 serous carcinoma	IVB	Primodiagnostic	non	*na*	9.84	5.21	12.2
LB-9195	71	F	grade 3 serous carcinoma	IVB	Primodiagnostic	yes	*na*	7.96	6.06	8.29
LB-9244	75	F	grade 3 serous carcinoma	IIIc	Primodiagnostic	yes	*na*	2.23	4.84	9.71
LB-9370	38	F	grade 3 serous carcinoma	IIIc	Primodiagnostic	no	*na*	13.3	41.4	18.7
LB-9370	38	F	grade 3 serous carcinoma	IIIc	Primodiagnostic	no	*na*	9.86	48.5	35.1
LB-9274	72	F	grade 3 serous carcinoma	IIIc	Primodiagnostic	yes	*na*	5.48	66.6	75.2
LB-9416	80	F	grade 3 serous carcinoma	IIIc	Primodiagnostic	no	*na*	8.09	4.64	16.1
LB-9329	73	F	grade 3 serous carcinoma	IIIc	Primodiagnostic	yes	*na*	9.25	18.2	31.2
LB-9460	61	F	grade 2 endometrioid adenocarcinoma + low grade serous carcinoma	IIIb	Primodiagnostic	no	23.3	2.74	*na*	*na*
LB-6119	44	F	grade 3 serous carcinoma	IIIc	Primodiagnostic	no	21.8	3.6	*na*	*na*
LB-9227	58	F	grade 3 serous carcinoma	IIIc	Primodiagnostic	no	29.5	3.6	*na*	*na*

Supplementary Table 3: Description of the cohort of patients with Covid19 disease
na = information unavailable
non-ICU = sample retrieved fom patient not hospitalized in intensive care unit
pre-ICU = sample retrieved from patient before hospitalization in intensive care unit
ICU = sample retrieved from patient hospitalized in intensive care unit

COVID19 patient	sample	% HELIOS+ in blood non naive CD8	age patient at blood sampling	gender	hospitalization stage
LB-9310	1	14	62	M	pre-ICU
LB-9310	2	13.3	62	M	ICU
LB-9319	1	6.35	74	F	non-ICU
LB-9319	2	11	74	F	non-ICU
LB-9395	1	73.9	63	M	pre-ICU
LB-9395	2	18.9	63	M	ICU
LB-9361	1	29.7	68	*na*	pre-ICU
LB-9361	2	31.3	68	*na*	ICU
LB-9309	1	28.1	27	M	non-ICU
LB-9309	2	15.8	27	M	non-ICU
LB-9342	1	20.2	50	*na*	pre-ICU
LB-9342	2	14.2	50	*na*	ICU
LB-9313	1	8.12	59	M	ICU
LB-9333	1	38.1	39	M	non-ICU
LB-9332	1	10.7	70	F	ICU
LB-9314	1	5.77	59	F	non-ICU
LB-9303	1	43	29	M	non-ICU
LB-9340	1	14.3	68	M	non-ICU
LB-9367	1	6.55	59	M	ICU
LB-9360	1	32.9	58	*na*	non-ICU
LB9322	1	23.6	51	M	non-ICU
LB-9359	1	21.5	47	*na*	non-ICU
LB-9325	1	23.9	50	M	non-ICU
LB-9358	1	11.3	61	*na*	non-ICU
LB-9404	1	9.68	80	M	ICU
LB-9363	1	8.16	63	*na*	non-ICU
LB-9351	1	19.3	60	*na*	ICU
LB-9316	1	11	85	M	ICU
LB-9343	1	17.6	41	*na*	non-ICU
LB-9405	1	7.49	68	M	ICU
LB-9384	1	41.6	42	M	non-ICU
LB-9383	1	14.2	65	M	ICU
LB-9387	1	4.69	68	M	ICU
LB-9381	1	11.3	73	M	non-ICU
LB-9385	1	2.11	*na*	*na*	*na*
LB-9375	1	14.9	42	F	non-ICU
LB-9382	1	12.8	53	M	non-ICU
LB-9377	1	41.6	50	M	non-ICU
LB-9386	1	32.5	53	M	ICU
LB-9379	1	14.6	67	M	non-ICU
LB-9380	1	11.4	*na*	*na*	na
LB-9376	1	13.1	42	M	non-ICU
LB-9389	1	20.7	25	F	non-ICU
LB-9388	1	24.4	58	M	non-ICU
LB-9398	1	5.27	68	M	ICU
LB-9393	1	17.5	72	F	ICU
LB-9399	1	3.27	63	F	ICU
LB-9391	1	51.1	*na*	*na*	*na*
LB-9400	1	39.4	63	M	ICU
LB-9402	1	7.95	81	M	ICU
LB-9403	1	57.1	55	M	ICU
LB-9392	1	27.8	47	M	non-ICU
